# Wuhan Sequence-Based Recombinant Antigens Expressed in *E. coli* Elicit Antibodies Capable of Binding with Omicron S-Protein

**DOI:** 10.3390/ijms25169016

**Published:** 2024-08-20

**Authors:** Ekaterina Evtushenko, Ekaterina Ryabchevskaya, Angelina Kovalenko, Dmitriy Granovskiy, Marina Arkhipenko, Yuri Vasiliev, Nikolai Nikitin, Olga Karpova

**Affiliations:** 1 Department of Virology, Faculty of Biology, Lomonosov Moscow State University, Moscow 119234, Russia; eryabchevskaya@gmail.com (E.R.); angelina-kovalenko94@mail.ru (A.K.); dgran98@gmail.com (D.G.); armar74@mail.ru (M.A.); nikitin@mail.bio.msu.ru (N.N.); karpovaov@my.msu.ru (O.K.); 2 R-Pharm, Moscow 119421, Russia; ym.vasilev@rpharm.ru

**Keywords:** SARS-CoV-2, recombinant protein vaccine, Omicron variant, plant virus-based adjuvant

## Abstract

The development of cross-reactive vaccines is one of the central aims of modern vaccinology. Continuous mutation and the emergence of new SARS-CoV-2 variants and subvariants create the problem of universal coronavirus vaccine design. Previously, the authors devised three recombinant coronavirus antigens, which were based on the sequence collected in 2019 (the Wuhan variant) and produced in an *E. coli* bacterial expression system. The present work has shown, for the first time, that these recombinant antigens induce the production of antibodies that clearly interact with produced in CHO full-length S-protein of the Omicron variant. The immunogenicity of these recombinant antigens was studied in formulations with different adjuvants: Freund’s adjuvant, Al(OH)_3_ and an adjuvant based on spherical particles (SPs), which are structurally modified plant virus. All adjuvanted formulations effectively stimulated Omicron-specific IgG production in mice. These universal coronavirus antigens could be considered the main component for the further development of broad-spectrum coronavirus vaccines for the prevention of SARS-CoV-2 infection. The present work also provides evidence that the synthetic biology approach is a promising strategy for the development of highly cross-reactive vaccines. Moreover, it is important to note that the bacterial expression system might be appropriate for the production of antigenically active universal antigens.

## 1. Introduction

COVID-19 is a severe infection. It has a zoonotic origin and is caused by the SARS-CoV-2 virus (family *Coronaviridae*, genus *Betacoronavirus*) [1]. Despite the fact that licensed vaccines have demonstrated good safety and protectiveness, their efficacy began to decline with the appearance of new SARS-CoV-2 genetic variants, with mutations mainly affecting the S-protein [2,3]. New variants and subvariants of SARS-CoV-2 are continually emerging. Just recently, the newest subvariant of Omicron, “Pirola”, was described [4,5]. This is evidence that it is particularly important to develop vaccines that are powerful regardless of the mutations arising in the main virus antigen. It is also important to test their efficacy in relation to various SARS-CoV-2 variants, including newly emerging ones [6].

The authors developed a COVID-19 vaccine candidate comprised of spherical particles (SPs) and three recombinant antigens, Co1, PE and CoF (3AG), which are expressed in *E. coli* cells [7,8]. SPs are a novel and promising plant virus-based adjuvant [9]. The amino-acid sequence of the Wuhan variant SARS-CoV-2 S-protein served as the basis for the design of these recombinant antigens. In a previous study, SPs’ adjuvant potential in a vaccine candidate against SARS-CoV-2 was tested. SPs enhanced the immunogenicity of 3AG. Furthermore, sera obtained from animals immunised with SPs + 3AG demonstrated neutralisation activity against SARS-CoV-2 in vitro [7]. The vaccination of hamsters with SPs + 3AG led to a reduction in the severity of pneumonia symptoms caused by SARS-CoV-2 [8]. The Wuhan variant of SARS-CoV-2 was used for protectiveness evaluation in both studies [7,8].

The present study assessed the applicability of 3AG-based vaccine candidates for protection against the currently circulating variant, Omicron. The cross-reactivity of sera obtained from animals immunised with the Wuhan sequence-based 3AG to the S-protein of Omicron variant SARS-CoV-2 was tested by means of immunoblot analysis and indirect ELISA. The 3AG formulations with various adjuvants (SPs-based adjuvant, complete Freund’s adjuvant and Al(OH)_3_) were used for the vaccination of animals and the sera titres of Omicron-specific total IgG were measured.

## 2. Results

**Figure 1:**
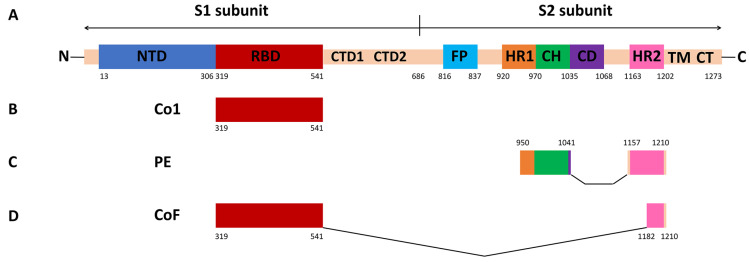
Linear diagram of the full-length SARS-CoV-2 spike protein domain structure (**A**) and coronavirus recombinant antigens Co1 (**B**), PE (**C**) and CoF (**D**). Coordinates of amino acid residues included in the recombinant antigens are indicated. Domains are coloured according to the coordinates indicated in Sun et al. (2021) [11]. Diagrams are not to scale. NTD—N-terminal domain; RBD—receptor-binding domain; CTD1 and CTD2—C-terminal domains 1 and 2; FP—fusion protein; HR1 and HR2—heptad repeats 1 and 2; CH—central helix; CD—connector domain; TM—transmembrane domain; CT—cytoplasmic tail.

Co1, PE and CoF are recombinant proteins that are produced in *E. coli* and that have previously been demonstrated to be promising coronavirus antigens [7,8]. These proteins represent fragments of SARS-CoV-2 S-protein (Figure 1A) corresponding to the sequence of the SARS-CoV-2 isolate Wuhan-Hu-1 (GenBank YP_009724390.1), collected in December 2019. A graphical overview of S-protein fragments incorporated into Co1, PE and CoF is shown in Figure 1. Co1 represents the RBD-domain (319–541 a.a.) (Figure 1B), while PE represents S-protein S2-subunit fragments 950–1041 a.a. (including part of HR1 and CH domains) and 1157–1210 a.a. (including the HR2 domain), which are highly conserved among betacoronaviruses (Figure 1C). CoF is a fusion of Co1-protein and a highly conserved protective epitope EIDRLNEVAKNLNESLIDLQELGKYEQYI (1182–1210 a.a.) (Figure 1D); antibodies specific to this epitope were previously isolated from the blood sera of humans with SARS pneumonia and were shown to have protective properties [10].

**Figure 2:**
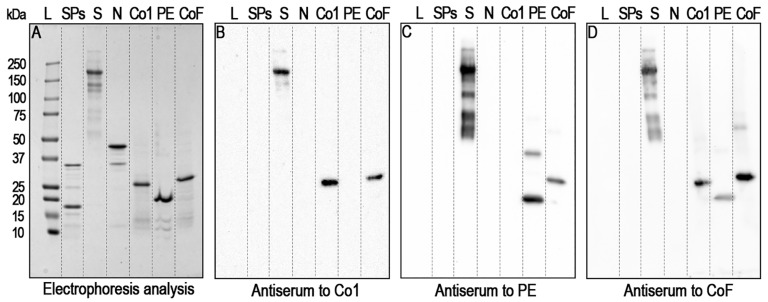
Interaction of recombinant S-protein of the Omicron variant of SARS-CoV-2 with mice polyclonal antisera specific to the RBD-domain and/or S2-subunit fragments of the Wuhan variant of SARS-CoV-2. L—protein molecular weight markers ladder (molecular weights, in kDa, are indicated on the left). S and N—SARS-CoV-2 B.1.1.529/Omicron recombinant S-protein and N-protein, respectively. Co1, PE, CoF—recombinant coronavirus antigens, which are expressed in the *E. coli* system. Co1 and PE represent SARS-CoV-2 isolate Wuhan-Hu-1 RBD-domain and conserved among betacoronaviruses S2-subunit fragments, respectively. CoF is a fusion of Co1-protein with a highly conserved epitope of S2-subunit. An 8–20% SDS-PAGE electrophoresis analysis, staining by Coomassie G-250 (**A**). Western blot analysis with primary polyclonal antisera specific to Co1 (1:3000) (**B**), PE (1:3000) (**C**) or CoF (**D**) (1:3000). Anti-mouse IgG HRP-conjugate was used as secondary antibodies in a dilution of 1:10,000.

The main aim of the present work was to assess whether these antigens would induce the production of antibodies bounding the S-protein of the heterologous variant Omicron. Firstly, the ability of polyclonal antisera collected from animals immunised with each of the antigens, Co1, PE or CoF, to recognise recombinant S-protein of SARS-CoV-2 B.1.1.529/Omicron was analysed using Western blot analysis (Figure 2). Co1, PE, CoF and SPs, as well as the recombinant N-protein of SARS-CoV-2 B.1.1.529/Omicron, served as positive or negative controls. All proteins used were visualised by means of electrophoresis analysis (Figure 2A). Omicron S-protein was shown to be recognised not only by antisera specific to proteins PE and CoF containing conserved sequences (Figure 2C,D) but also by antiserum specific to the Co1 antigen, containing only the RBD domain sequence (Figure 2B). Notably, the anti-Co1 serum recognised mainly the full-size form of S-protein (Figure 2B), corresponding to the major electrophoretic band (Figure 2A); meanwhile, the antiserum specific to the PE, which consisted only of S2-fragments, clearly recognised the set of S-protein fragments and isoforms with lower or higher electrophoretic mobility (Figure 2C). The antiserum specific to CoF also recognised the set of S-protein fragments and isoforms (Figure 2D); added to the fact that CoF bounded with anti-PE serum (Figure 2C) and PE bounded with anti-CoF serum (Figure 2D), this suggests that there is functional activity of the HR2 epitope within CoF.

**Figure 3:**
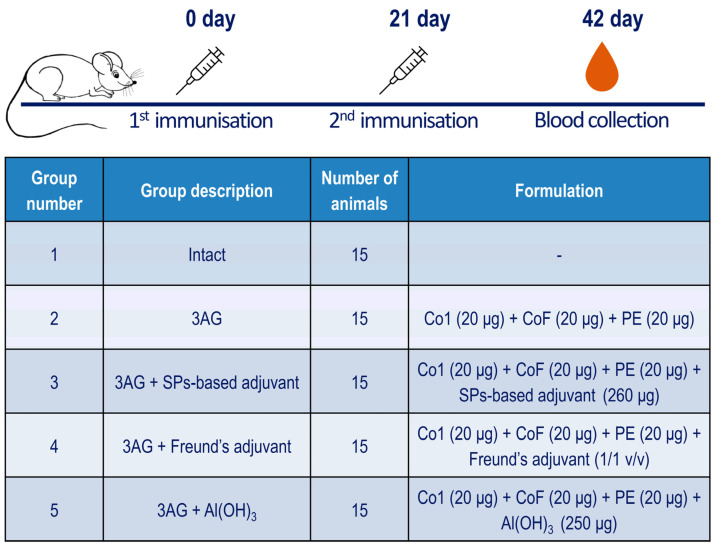
Immunisation schedule and description of mice groups involved in the experiment. Mice from groups 2–5 were immunised intramuscularly with a corresponding formulation containing 60 µg of 3AG (20 µg each). All samples administered were prepared in PBS; the final volume was 0.26 mL. In the case of the formulation with Freund’s adjuvant, the 3AG solution was prepared in half of the final volume and mixed with an equal amount of Freund’s adjuvant immediately prior to the immunisation. 3AG—the mixture of the three coronavirus recombinant antigens Co1, PE and CoF in equal mass ratio.

The ability of 3AG to induce the production of antibodies that would be able to interact with the S-protein of the Omicron variant was studied in a mouse model. As well as the intact control group (group 1), there were four experimental groups of mice that were immunised twice intramuscularly, with a 3-week interval between the immunisations. Mice of group 2 were injected with 60 μg of 3AG (the mixture of 20 μg of Co1, 20 μg of PE and 20 μg of CoF antigens) formulated in PBS solution with no additional components. Mice of the other groups were injected with the same amount of 3AG but in composition with one of the following adjuvants: SPs-based adjuvant (group 3), Freund’s adjuvant (group 4) or Al(OH)_3_ (group 5). The immunisation schedule and a summary description of the groups are presented in Figure 3.

**Figure 4:**
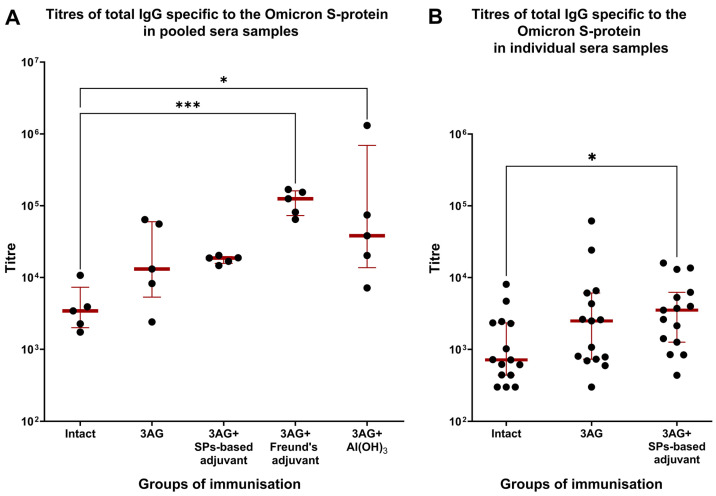
Comparison of the titres of total IgG specific to the S-protein of SARS-CoV-2 variant Omicron in mice sera obtained from intact animals and animals immunised with a mixture of recombinant antigens Co1, PE and CoF (3AG), either individually or in composition with various adjuvants. Mice were immunised intramuscularly twice, with a 21-day interval between immunisations. The scheme of the study is presented in Figure 3. Titres were evaluated by indirect ELISA. For all groups, titres of sera pools were detected (**A**) and, for groups with 3AG and 3AG + SPs-based adjuvant, titres of individual mice sera were subjected to additional analysis (**B**). Antigen concentration on the microplate was 5 µg/mL for evaluation of titres in pooled sera, and 2.5 µg/mL for evaluation of titres in individual mice sera. ●—titres of sera pool or individual mice serum; ▬—median; **I**—interquartile range. The Kruskal–Wallis test with a post hoc Dunn’s test was used for multiple comparisons of titres from each group with the titres from the group of intact animals. * *p* < 0.05, *** *p* < 0.001. The complete data on sera pool titres and individual mice sera titres are presented in Table S2 and Table S3, respectively.

The general condition of the animals during the immunisation period was monitored. There were no animal deaths and no signs of abnormality. Three weeks after the second immunisation, blood was collected. For each group, five pools were prepared by mixing an equal volume of sera collected from three different mice of the same group. In these pools, levels of anti-Omicron S-protein total IgG were measured by indirect ELISA (Figure 4A, Table S1). Pools prepared from group 1 (intact) were also titrated on the N-protein (Table S2) in order to assess the level of background immunity to SARS-CoV-2. The anti-Omicron S-protein total IgG titres in sera taken from the experimental groups were compared with those of group 1. A statistically significant difference was identified between group 1 (median—3421) and groups 4 (median—124,915) and 5 (median—38,106). This demonstrates that 3AG in composition with Freund’s adjuvant (group 4) or Al(OH)_3_ (group 5) elicits the production of Omicron-specific IgG.

For groups 2 and 3, for which no significant difference from intact animals was detected by analysis of sera pool titres, total IgG titres of individual sera were also assessed (Figure 4B, Table S3). This evaluation of individual serum titres revealed a statistically significant difference between the intact group (median—715) and group 3 (median—3516). This indicates that the composition of 3AG with SPs-based adjuvant effectively elicits the production of IgG that can interact with the S-protein of the Omicron variant. No statistically significant difference between group 1 and group 2 (median—2491), the mice of which had been immunised with 3AG and no adjuvants, was detected.

## 3. Discussion

Here, recombinant proteins Co1, PE and CoF, based on the Wuhan variant SARS-CoV-2 sequence and produced in *E. coli*, were characterised as potential antigens for a universal vaccine. Three different mice sera specific to Co1, PE or CoF were clearly demonstrated to interact with the S-protein of the heterologous Omicron variant of SARS-CoV-2, using a Western blot analysis. Unlike PE and CoF, which contained conserved sequences from the HR2 region of the S-protein, Co1 was represented only by the sequence corresponding to RBD. In a study by Rahbar et al. (2022), RBD-based recombinant antigen was shown to be recognised by serum antibodies from individuals who had recovered from COVID-19 caused by the Delta variant of SARS-CoV-2, for which there are two critical mutations in RBD [12]. Here, the ability of anti-Co1 serum to interact with the S-protein of the Omicron variant, for which there are 15 essential mutations in RBD, was shown.

The ability of 3AG formulations to induce a cross-reactive immune response to Omicron’s S-protein was studied in a mouse model. A statistically significant difference between IgG titres in the sera of mice immunised with 3AG (without any adjuvant) and intact mice was not revealed. This is explainable since recombinant proteins usually have low immunogenicity by themselves and their application requires the presence of a suitable adjuvant [13,14]. All adjuvanted 3AG formulations were reliably demonstrated to elicit anti-Omicron S-protein IgG. As expected, immune response in a group of mice immunised with the 3AG in composition with complete Freund’s adjuvant, which was used as a positive control, was high, but this adjuvant is inappropriate for use in vaccines [13]. Two other compositions, 3AG + SPs-based adjuvant and 3AG + Al(OH)_3_, could be considered to be appropriate broad-spectrum vaccine candidates, although aluminium hydroxide is known as an adjuvant that induces a strong humoral Th2-biased immune response [15,16]. Nevertheless, a number of studies have suggested that, for protection against betacoronaviruses, the induction of a well-balanced Th1/Th2—or even a Th1-biased—immune response is preferable [17,18,19,20]. Meanwhile, SPs could stimulate both a humoral and a cellular branch of immune responses [9]. SPs’ composition with 3AG has previously been shown to elicit an immune response biased to anti-PE and anti-CoF antibodies. Titres of IgG1 and IgG2a isotypes were comparable, which testifies in favour of the induction of a balanced immune response. However, it should be noted that the dose of the vaccine candidate and the route of the administration differed in that study [7]. We suppose that developed antigens could be a part of vaccine candidates against different variants of SARS-CoV-2, including Omicron, and possibly even against another pandemic and pre-pandemic betacoronaviruses. These data provide evidence that the synthetic biology approach and *E. coli* expression system are more than feasible for the development of highly cross-reactive vaccines. Such an approach could be used to counter future waves of SARS-CoV-2 and improve pandemic preparedness.

## 4. Materials and Methods

### 4.1. Recombinant Antigens and SPs Production

Recombinant antigens Co1, PE and CoF, and SPs, were obtained as described previously [7,8].

### 4.2. Immunisation of Mice and Sera Collection

The study included five groups of BALB/c female mice (4–5 weeks old; *n* = 15). The animals of group 1 were intact, while those of groups 2–5 were immunised with mixtures of three coronavirus recombinant antigens—3AG (Co1, PE and CoF). Each dose contained 60 μg of 3AG (20 μg each), either without any adjuvant (group 2) or in combination with various adjuvants: 260 μg of SPs-based adjuvant (group 3), 1:1 (*v*/*v*) Freund’s adjuvant complete (F-5881, Sigma-Aldrich, St. Louis, MO, USA) (group 4) or 250 μg of Al(OH)_3_ Alhydrogel (vac-alu-250, InvintoGen™, Waltham, MA, USA) (group 5). All administered formulations were prepared in PBS. Where Freund’s adjuvant was administered, the 3AG solution was prepared in half of the final volume and mixed with an equal amount of Freund’s adjuvant immediately prior to the immunisation. The dose volume was 0.26 mL per animal. Animals were immunised twice, at 21-day intervals, intramuscularly in the thigh muscle. Blood was collected by decapitation 21 days after the second immunisation. The scheme of the study is presented in the “Results” Section (Figure 3). Within each group, five pools were prepared by mixing an equal volume of sera samples collected from three individual mice.

To obtain the sera specific only to the one protein (anti-Co1, anti-CoF or anti-PE), three different mice were injected intraperitoneally with 10 μg of the corresponding individual antigen Co1, CoF or PE. Samples were prepared in 0.1 mL of PBS and mixed with 0.1 mL of Freund’s adjuvant (F-5506, Sigma-Aldrich, St. Louis, MO, USA); the complete one was used for the first immunisation and the incomplete one for the two subsequent boosters, which were carried out in two-week intervals. Serum from each individual mouse injected with Co1, CoF or PE was used in Western blot analysis as anti-Co1, anti-CoF or anti-PE serum, respectively.

### 4.3. Ethical Statement

The methods used in experiments with mice were performed in accordance with relevant guidelines and regulations and approved by the Commission on Bioethics of the Center for Preclinical Studies of the Institute for Translational Medicine and Biotechnology of the I.M. Sechenov First Moscow State Medical University of the Ministry of Health of the Russian Federation (Sechenov University) (Protocol 111 dated 21 October 2022).

### 4.4. Western Blot Analysis

Western blot analysis was performed as described earlier [7]. As primary antibodies, one of the polyclonal mice antisera specific to Co1, PE or CoF was used in a 1:3000 dilution. Secondary anti-mouse IgG HRP-conjugate (115-035-003, Jackson ImmunoResearch Laboratories, Inc., West Grove, PA, USA) was used in a dilution of 1:10,000. The WesternBright ECL chemiluminescent substrate (Advansta Inc., San Jose, CA, USA) was used and the signal was detected using the ChemiDoc XRS+ gel documentation system (Bio-Rad Laboratories, Inc., Hercules, CA, USA).

### 4.5. ELISA

ELISA was performed, according to the protocol described earlier [7]. Recombinant S-protein (#ab290830, Abcam, Cambridge, UK) or recombinant N-protein (#40588-V07E34, Sino Biological US Inc., Beijing, China) of SARS-CoV-2 B.1.1.529/Omicron was used as an antigen. For coating 96-well plates, N-protein was used at a concentration of 8 μg/mL. The concentration of S-protein was 5 μg/mL for detecting sera pool titres and 2.5 μg/mL for detecting individual sera titres. Anti-mouse total IgG HRP conjugate (#ab6728, Abcam, Cambridge, UK) was used in a dilution of 1:10,000. Sera on the N-protein and all sera pools were titrated in 3-fold serial dilutions, starting from 1:30. For the titration of individual mice sera samples on the S-protein, the starting dilution was 1:300. The serum titre was defined as the reciprocal of the serum dilution at which A_450_ was equal to the mean of the block + 3SD.

### 4.6. Statistical Analysis

For a comparison of the IgG titres’ values between different groups, the Kruskal–Wallis test, with a post hoc Dunn’s test, was used. *p*-values of less than 0.05 were considered significant. The statistical processing of the results and the graph plotting were carried out using GraphPadPrism 9.1.0. (GraphPad Software, La Jolla, San Diego, CA, USA).

## Data Availability

All the relevant data are provided in this paper and in the Supporting Materials.

## Supplementary Materials

The following supporting information can be downloaded at https://www.mdpi.com/article/10.3390/ijms25169016/s1.
