# Supplementary material for: Wuhan Sequence-Based Recombinant Antigens Expressed in E. coli Elicit Antibodies Capable of Binding with Omicron S-Protein
Source: Int J Mol Sci. 2024 Aug 20;25(16):9016. doi: 10.3390/ijms25169016 (PMC11354337; doi:10.3390/ijms25169016)
Supplement: Supplementary file 1 [file ijms-25-09016-s001.zip › Table S1.pdf]

Identification number of pool	Total IgG titre				
	Group 1 (intact control)	Group 2 (3AG)	Group 3 (3AG + SPs-based adjuvant)	Group 4 (3AG + Freund's adjuvant)	Group 5 (3AG + $Al(OH)_3$)
Pool 1	10,713	63,967	14,654	81,079	7,152
Pool 2	3,897	2,401	18,783	64,428	20,244
Pool 3	2,259	8,237	16,795	124,915	74,132
Pool 4	3,421	13,116	20,298	168,180	38,106
Pool 5	1,732	55,525	18,657	153,610	1,309,211
**Median**	**3,421**	**13,116**	**18,657**	**124,915**	**38,106**

Table S1: Total IgG titres to the recombinant S-protein of SARS-CoV-2 B.1.1.529/Omicron (#ab290830, Abcam, Cambridge, UK) in pools of mice sera. Group 1 consisted of intact mice (control). Groups 2-5 were immunised intramuscularly twice (days 0, 21), either with 60 μg of 3AG (coronavirus recombinant antigens Co1, PE and CoF – 20 μg each) (group 2), or with the same amount of 3AG in compositions with one of the following adjuvants: SPs-based adjuvant (group 3), Freund's adjuvant (group 4) or $Al(OH)_3$ (group 5). The scheme of the study is presented in Figure 3. All samples administered were in PBS in a total volume of 0.26 ml. Blood was collected after the second immunisation on the 42nd day of the experiment. Each pool consisted of a mixture of an equal volume of sera collected from three mice of the same group. Titres were evaluated by in-direct ELISA. The concentration of S-protein on the microplate was 5 μg/ml; anti-mouse IgG HRP conjugate (ab6728) was used as secondary antibodies.
