# Supplementary material for: Wuhan Sequence-Based Recombinant Antigens Expressed in E. coli Elicit Antibodies Capable of Binding with Omicron S-Protein
Source: Int J Mol Sci. 2024 Aug 20;25(16):9016. doi: 10.3390/ijms25169016 (PMC11354337; doi:10.3390/ijms25169016)
Supplement: Supplementary file 1 [file ijms-25-09016-s001.zip › Table S2.pdf]

Titres of mice sera titrated on N-protein		
Group description	Identification number of serum/pool	Titre IgG total
Sera collected before the start of experiment	1	2,207
	2	1,749
	3	1,913
	4	2,294
	5	5,551
	**Median**	**2,207**
Pools of group 1 (intact control)	Pool 1	669
	Pool 2	1,516
	Pool 3	634
	Pool 4	760
	Pool 5	738
	**Median**	**738**

Table S2: Total IgG titres to the recombinant N-protein of SARS-CoV-2 B.1.1.529/Omicron (#40588-V07E34, Sino Biological US Inc., Beijing, China) in pools of group 1 (intact) mice sera collected on the 42nd day of the experiment. Titres were evaluated by indirect ELISA. Concentration of N-protein on the microplate was 8 μg/ml; anti-mouse IgG HRP conjugate (#ab6728, Abcam, Cambridge, UK) was used as secondary antibodies.
