# Supplementary material for: Wuhan Sequence-Based Recombinant Antigens Expressed in E. coli Elicit Antibodies Capable of Binding with Omicron S-Protein
Source: Int J Mol Sci. 2024 Aug 20;25(16):9016. doi: 10.3390/ijms25169016 (PMC11354337; doi:10.3390/ijms25169016)
Supplement: Supplementary file 1 [file ijms-25-09016-s001.zip › Table S3.pdf]

Identification number of mouse	Total IgG titre		
	Group 1 (intact control)	Group 2 (3AG)	Group 3 (3AG + SPs-based adjuvant)
1	300*	6,092	13,006
2	4,690	2,586	1,413
3	613	300*	1,261
4	442	4,322	3,516
5	1,021	732	2,610
6	2,295	2,491	15,961
7	8,068	804	3,970
8	2,441	595	437
9	2,337	61,425	5,287
10	715	24,138	846
11	721	1,071	840
12	440	695	2,132
13	300*	785	13,592
14	621	2,619	6,240
15	300*	6,556	3,727
**Median**	**715**	**2,491**	**3,516**

Table S3: Total IgG titres to the recombinant S-protein of SARS-CoV-2 B.1.1.529/Omicron (#ab290830, Abcam, Cambridge, UK) in individual mice sera. Group 1 consisted of intact mice (control). Groups 2 and 3 were immunised intramuscularly twice (days 0, 21), either with 60 μg of 3AG (coronavirus recombinant antigens Co1, PE and CoF – 20 μg each) (group 2), or with the same amount of 3AG in compositions with SPs-based adjuvant (group 3). The scheme of the study is presented in Figure 3. All samples administered were in PBS in a total volume of 0.26 ml. Blood was collected after the second immunisation on the 42nd day of the experiment. Titres were evaluated by indirect ELISA. The concentration of S-protein on the microplate was 2.5 μg/ml; anti-mouse IgG HRP conjugate (ab6728) was used as secondary antibodies. (*) Serum titre that was taken to be 300, since an A_{450} in the starting dilution of 1:300 was below the mean value of the block + 3SD.
